# Determination of Arsenic Species in *Ophiocordyceps sinensis* from Major Habitats in China by HPLC-ICP-MS and the Edible Hazard Assessment

**DOI:** 10.3390/molecules23051012

**Published:** 2018-04-26

**Authors:** Lian-Xian Guo, Gui-Wei Zhang, Jia-Ting Wang, Yue-Ping Zhong, Zhi-Gang Huang

**Affiliations:** 1 Dongguan Key Laboratory of Environmental Medicine, School of Public Health, Guangdong Medical University, Dongguan 523808, China; glx525@gdmu.edu.cn (L.-X.G.); Vivian_jtw@163.com (J.-T.W.); ZYP13415680421@live.com (Y.-P.Z.); 2 Shenzhen Academy of Metrology and Quality Inspection, Shenzhen 518000, China; zhguiw98@163.com

**Keywords:** *Ophiocordyceps sinensis*, arsenic speciation, risk assessment, HPLC-ICP-MS

## Abstract

This study sought to determine the concentration and distribution of arsenic (As) species in *Ophiocordyceps sinensis* (*O. sinensis*), and to assess its edible hazard for long term consumption. The total arsenic concentrations, measured through inductively coupled plasma mass spectrometry (ICP-MS), ranged from 4.00 mg/kg to 5.25 mg/kg. As determined by HPLC-ICP-MS, the most concerning arsenic species—AsB, MMA^V^, DMA^V^, As^V^, and As^Ш^—were either not detected (MMA^V^ and DMA^V^) or were detected as minor As species (AsB: 1.4–2.9%; As^V^: 1.3–3.2%, and As^Ш^: 4.1–6.0%). The major components were a cluster of unknown organic As (uAs) compounds with As^Ш^, which accounted for 91.7–94.0% of the As content. Based on the H_2_O_2_ test and the chromatography behavior, it can be inferred that, the uAs might not be toxic organic As. Estimated daily intake (*EDI)*, hazard quotient (*HQ*), and cancer risk (*CR*) caused by the total As content; the sum of inorganic As (iAs) and uAs, namely i+uAs; and iAs exposure from long term *O. sinensis* consumption were calculated and evaluated through equations from the US Environmental Protection Agency and the uncertainties were analyzed by Monte-Carlo Simulation (MCS). *EDI*_total As_ and *EDI*_i+uAs_ are approximately ten times more than *EDI*_iAs_; *HQ*_total_ _As_ and *HQ*_i+u__As_ > 1 while *HQ*_i__As_ < 1; and *CR*_total As_ and *CR*_i+uAs_ > 1 × 10^−4^ while *CR*_iAs_ < 1 × 10^−4^. Thus, if the uAs is non-toxic, there is no particular risk to local consumers and the carcinogenic risk is acceptable for consumption of *O. sinensis* because the concentration of toxic iAs is very low.

## 1. Introduction

**Figure 1:**
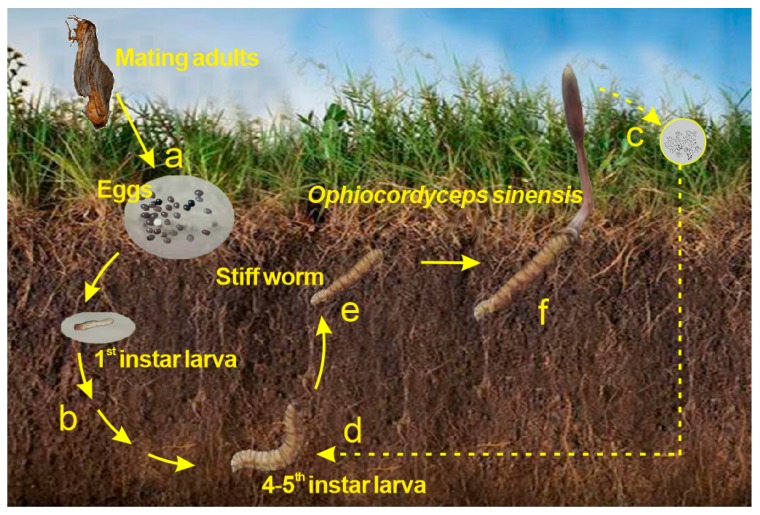
Life history of *Ophiocordyceps sinensis* (*O. sinensis*). (a) The eggs of the host *Thitarodes* insect, which are scattered on the grassland, incubate; (b) The host larvae safely reside in the soil throughout the long-lasting larval stage; (c) The ascospores erupt from mature fruiting bodies of *O. sinensis*; (d) The 4–5th instar larvae may be infected by the infective conidia of the *O. sinensis* fungus in the soil; (e) The caterpillar-shaped sclerotium (winter-worm) is formed; (f) The stroma germinates out of the head capsule and the mature *O. sinensis* (summer-grass-winter-worm) is formed.

**Figure 2:**
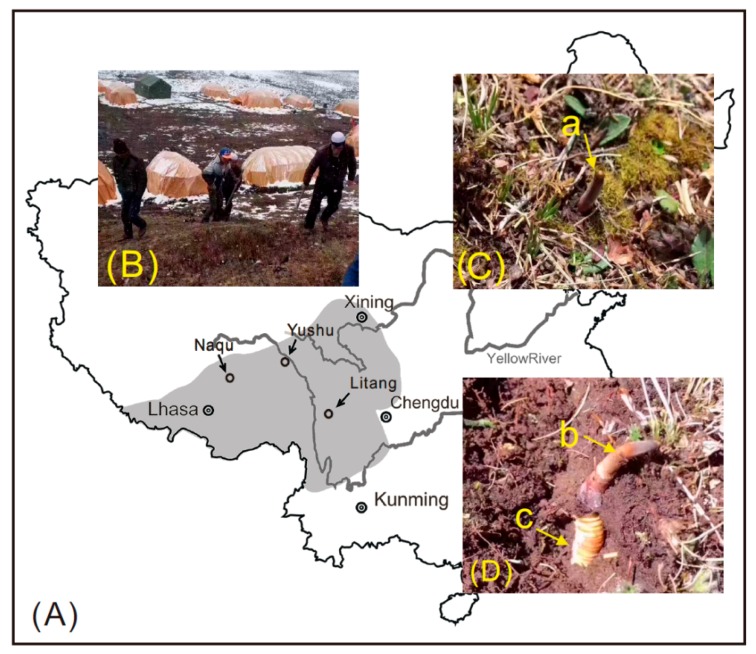
The producing area of *Ophiocordyceps sinensis* in China and sampling details of this study. (**A**) Schematic map illustrating the sampling sites in the Qinghai–Tibetan Plateau and its adjacent high-altitude areas. Litang (LT), Naqu (NQ), and Yushu (YS) were chosen as the sampling sites. (**B**) The grassland of the *O. sinensis* habitat. Pastoralists are encamped there to collect it. (**C**) The stroma (a) of *O. sinensis* emerged out of the ground. (**D**) *O. sinensis* in the soil, the yellow arrows pointing out its sclerotium (b) and stroma (c).

*Ophiocordyceps sinensis (O. sinensis)*, a mysterious entomogenous fungus distributed on the Qinghai–Tibet Plateau, is popularly referred to as winter-worm-summer-grass (Dong Chong Xia Cao in Chinese) [1,2] (Figure 1). *O. sinensis* has been utilized in China (Figure 2A) and surrounding countries for more than 2000 years as a rare functional food to promote health and to treat diverse chronic diseases. In 1993, *O. sinensis* became famous worldwide because Chinese female athletes broke several world records in running events at the National Games and the meritorious performances were later attributed (at least in part) to the consumption of this fungus [3]. Subsequently, modern pharmaceutical research has shown that its predominant functions are anti-tumor, anti-inflammatory, nephroprotective, antioxidant, antihyperglycemic, anti-apoptosis, immunoregulatory, and hepatoprotective [4,5]. Accordingly, these pronounced medicinal functions have resulted in a large demand for wild *O. sinensis* and have also increased the local prosperity of economically poor production areas around the Qinghai–Tibet Plateau and in adjacent countries. Some households earn as much as two-thirds of their income from the collection of *O. sinensis* (Figure 2B), and pastoralists are earning an income of a size unrecorded in their history [6,7]. However, concerns about human health have arisen since the CFDA (China Food and Drug Administration) revealed in 2016 that excessive Arsenic (As) content (total As: 4.4–9.0 mg/kg [8]) was detected in *O. sinensis*, a level five times the reference value of 1 mg/kg for total As in functional foods (GB16740-2014) [9]. Subsequently, the CFDA ordered that all the pilot work on *O. sinensis* as a functional food be discontinued on 26 February 2016 [10]. Moreover, numerous media outlets had arbitrarily declared that *O. sinensis* is a poison instead of a functional food. This assertion has caused a great uproar in the health food market and has seriously affected the *O. sinensis*-dependent economic chain [11].

Arsenic can be present in both organic and inorganic forms. The toxicity of As is dependent on its chemical form, with inorganic species (iAs), such as arsenite (As^Ш^) and arsenate (As^V^), being the most toxic [12]. Organic species are the metabolic products of iAs, such as monomethylarsonic acid (MMA^V^) and dimethylarsenic acid (DMA^V^), and are much less toxic than iAs to humans. Additionally, some other organic As complexes (arsenocholine, arsenobetaine, various arsenosugars and arsenolipids) are generally considered nontoxic [13], according to previous studies on As toxicity. However, recently, experimental results have documented the presence of trivalent intermediates, monomethylarsonous acid (MMA^Ш^) and dimethylarsinous acid (DMA^Ш^) in the urine of humans exposed to drinking water containing high levels of inorganic As [14]. These trivalent intermediates are structurally different from the pentavalent compounds and are more reactive and more carcinogenic [15,16,17]. More recently, the subsequent metabolic products of DMA^Ш^, sulfur-containing intermediary metabolites (dimethylmonothioarsinicacid, DMMTA^V^) [18], and several As containing hydrocarbons (AsHC 332, AsHC 360 and AsHC 444) were shown to high toxic effects on organisms [19]. The different toxicities of As species reinforce the importance of distinguishing its chemical form, as the total amount of As does not provide enough information about the toxicity of the analyzed sample. Therefore, it is incorrect to consider *O. sinensis* is toxic according to its total As content. Unlike previous reported arsenic accumulated mushrooms, which germinated on plant sourced media, *O. sinensis* only lives in *Thitarodes* larva of 4–5th instar. Inspired by the significantly higher organic As proportion in animal-sourced traditional Chinese medicine (TCM) compared to plant-sourced TCM [20], some scholars have proposed that due to larva-fungus complexity (Figure 2C,D), *O. sinensis* might contain a large proportion of organic As. Recently, researchers have provided evidence for the speculation that iAs species were minor (As^Ш^ and As^V^) or below the level of detection after examining the As speciation in *O. sinensis* through high-performance liquid chromatography-hydride generation-atomic fluorescence spectrometry (HPLC-HG-AFS). In addition, they found that the largest proportion of As was composed of an unknown organic As (uAs) species [21]. Because of the shortcomings of conventional HPLC-HG-AFS, in that research, they only discriminated four As species (MMA^V^, DMA^V^, As^V^ and As^Ш^) in *O.* *sinensis* samples [21]. Non-forming hydride species, such as AsB, important in biota samples, cannot be evaluated using this approach [22].

To further clarify the As speciation in *O. sinensis* and oral intake hazards in longtime consumption, the present study examined the total As content, including the most concerning As species (As^Ш^, As^V^ MMA^V^, DMA^V^, and AsB), in *O. sinensis* using the most commonly applied techniques: inductively coupled plasma mass spectrometry (ICP-MS) and anion exchange high-performance liquid chromatography–inductively coupled plasma mass spectrometry (HPLC-ICP-MS) [23,24]. Due to the extraordinary prices of the *O. sinensis* and chronic injury effects to organisms due to Arsenic consumption, a hazard assessment via experimental animal trials would be costly. Thus, in this study, we chose the most widely accepted model (Environment Protection Agency, EPA) of evaluation for health risks [25,26] to evaluate the potential non-carcinogenic risk and the probability of excess lifetime cancer risk due to As exposure from *O. sinensis* consumption. Additionally, Monte-Carlo Simulation (MCS) were employed to analyze the uncertainty. The health risk posed by *O. sinensis* consumption from exposure to total As, iAs, and i+uAs (iAs and uAs together) were comparatively estimated in this study. These contributions might be useful in efforts to revive the local *O. sinensis*-dependent economy in the Qinghai–Tibet Plateau and surrounding countries by providing a comprehensive edible hazard evaluation of *O. sinensis*.

## 2. Results and Discussion

### 2.1. Analytical Performances of the Proposed Method

Extraction efficiency for As speciation analysis was assessed based on a comparison between the total As concentration in 0.15 mol/L HNO_3_ extracts and concentrated HNO_3_ extracts using a microwave digestion system (see Section 3.2). The concentrations of the total As in the extracts through different extraction methods were quantitively consistent, with an extraction efficiency of 92.3–104% (the ratio of total As concentration in 0.15 mol/L HNO_3_ extracts to that in concentrated HNO_3_ extracts). This efficiency indicates that most As species were thoroughly extracted through the current method (see Section 3.2).

The analytical performances of the proposed ICP-MS, for total As content analysis, and HPLC-ICP-MS, for As species analysis, methods were validated by determining the linearity, limits of detection (LOD), and limits of quantification (LOQ) as shown in Table 1. The linear correlation coefficients, in the range of 0.5–500 μg L^−1^(for total As) and 0.2–300 μg L^−1^ (for As species), were greater than 0.9997. LOD of total As, AsB, DMA^V^, As^Ш^, MMA^V^, and As^V^ were 2.3, 1.1, 1.3, 1.0, 2.2, and 1.1 μg kg^−1^, respectively, And the LOQ were found to be in the range of 3.0–6.9 μg kg^−1^ for the total As content and the five arsenic species. The recoveries of the total As content were in the range 92.3−106.6% and the relative standard deviations (RSDs) were in the range 2.4~4.6%. The recoveries and RSDs of arsenic species were studied by spiking three concentration levels of the five arsenic species into the *O. sinensis* samples. The recoveries of arsenic species were in the range 86.3~111.7% and the RSDs were in the range 0.8~6.6%, as shown in Table 2. The analysis results of certified reference materials (CRM) using the same method were in good agreement with their certified values and the recoveries were 97.5–101% for total As analysis and 110.8% for iAs analysis as shown in Table 3.

### 2.2. Total Arsenic Concentration and Arsenic Species in O. sinensis Samples

**Figure 3:**
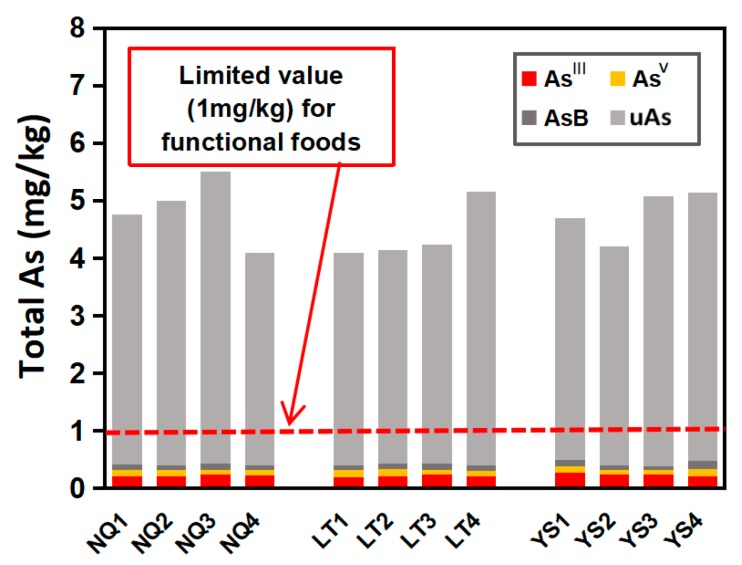
The concentration (mg/kg dry weight) of total As and As speciation detected in *O. sinensis*. Inorganic As (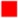 As^Ш^ and 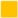 As^V^) are shown in red and yellow sections, and organic As (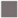 AsB and 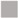 uAs) is shown in dark and light gray sections.

**Figure 4:**
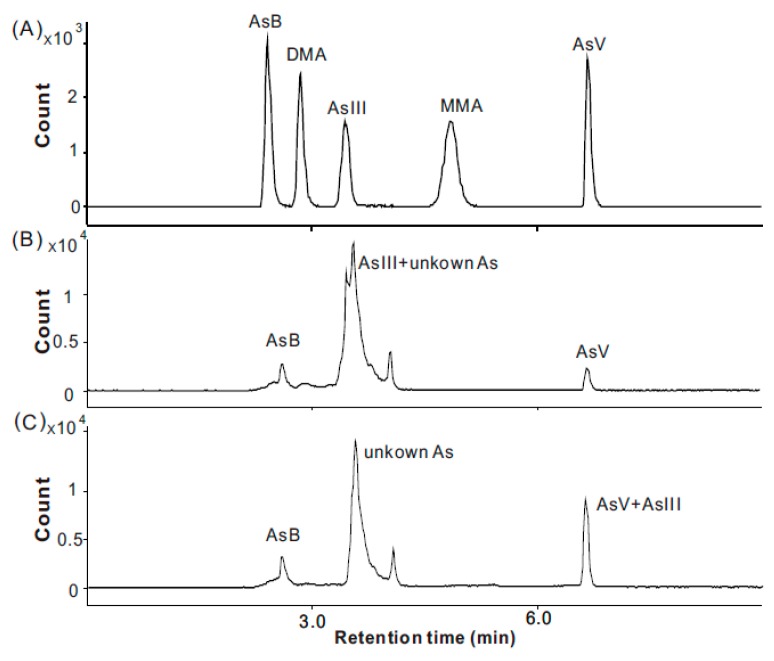
Chromatograms obtained in quantification by HPLC-ICP-MS. (**A**) A mix of standard samples of AsB, DMA^V^, As^Ш^, MMA, and As^V^, at 10 ppb of each arsenic species. (**B**) The extracts of sample NQ1. The As^Ш^ and unknown organic As peaks overlap. (**C**) Oxidation products of the extracts of NQ1. Any As^Ш^ is transformed into As^V^ when H_2_O_2_ is added to the extracts.

**Figure 5:**
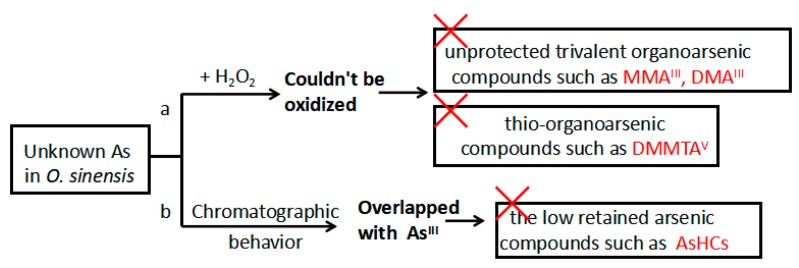
Inference on the toxicity of the unknown As detected in *O. sinensis.* (a) 1 mL of H_2_O_2_ was added to the extracts, and the unknown As could not be oxidized (Figure 4C). Thus, the unknown As cannot be the toxic MMA^Ш^, DMA^Ш^, or DMMTA^V^ which can be oxidized under treatment with H_2_O_2_. (b) The unknown peak presents a similar retention time with As^Ш^, indicating that it is not a low retention component, such as an As hydrocarbon (AsHC).

The total As content in *O. sinensis* ranged from 4.00 mg/kg to 5.25 mg/kg of dry mass (Table 4), which is consistent with that in the previous studies [21] that caused uneasiness and fear in consumers. The Chinese government, through the National Health and Family Planning Commission of the People’s Republic of China, established a reference value of 1 mg/kg for total As in functional foods (GB16740-2014) [9]. Thus, the total As content in *O. sinensis* exceeds the limit of As in functional foods in China (Figure 3). These data demonstrate that there is an urgent need to determine As speciation in *O. sinensis* samples, because the total amount of As does not provide sufficient toxicological information. In Figure 4B, the large peak area indicates that As^Ш^, which is generally considered most toxic, might be the major As species in *O. sinensis*, however, an H_2_O_2_ test proved that most of the As is not oxidized to As^V^ (Figure 4C) and hence the major overlapped peak cannot be the toxic As^Ш^. Thus, according to current chromatography conditions, an H_2_O_2_ test is necessary to minimize misidentification. The iAs, calculated according to the As^V^ content in Figure 4C, is relatively abundant compared to the total As content, in the range 6.0% to 8.3% (the amount of As^V^ in Figure 4C), but is small compared with the amount of organic As. Organic As species were predominant, and the percentage of total As content ranged from 91.7% to 94.0%. The two potential toxic organic As species, MMA^V^ and DMA^V^, were found to be negligible. Because the initial organic metabolites of iAs, MMA^V^, and DMA^V^ were trace in *O. sinensis* sample, it can be inferred that they were transformed into other organic arsenic metabolites in the organism such as AsB (which was also detected as a minority compound in these samples, ranging from 1.4% to 2.9%) and various unknown organic As species (uAs, a cluster of unknown compounds, with a retention time from 3.2 to 4 min, representing 89.0% to 92.3% of the of the total As content, Figure 4C). Based on previous studies, comparison of the retention time behavior of standards with that of the extracts upon changing pH of the mobile phase can ascertain the presence of an expected compound or give useful information on the nature of unknown compounds [27]. In this study, despite the lack of standards for the confirmation of uAs, based on the overlap with As^Ш^, organic As (oAs) species which have low retention under anion exchange HPLC-ICP-MS chromatograms, such as arsenolipids and AsHCs which were usually analyzed using reversed-phase HPLC-ICP-MS, can be excluded [28]. In addition, according to its stability under the H_2_O_2_ treatment, unprotected trivalent oAs species which can be oxidized, such as DMA^Ш^, MMA^Ш^, and thio-organoarsenic (DMMTA^V^), can also be excluded. Thus, due to its chemical characteristics, the uAs in *O. sinensis* cannot be the toxic oAs which have been discovered so far (Figure 5). The chromatography behavior indicates that it might be an arsenosugar(s), which are frequently reported to be co-eluted with DMA, AsB, MMA, and As^Ш^ under anion exchange HPLC-ICP-MS chromatograms [29].

### 2.3. Hazard Risk Assessment of Long-Term O. sinensis Consumption

Because the toxicity of As species differs [17], it is especially important to determine the chemical form of As in *O. sinensis* samples, and a health risk assessment should focus on toxic As species because of their carcinogenic potential rather than the total As content. Based on the As speciation analysis method of the latest Chinese national standard to determine As species in functional food (GB 16740-2014), in this work iAs and AsB were minor As components. Additionally, MMA^V^ and DMA^V^ were negligible, leaving the majority of As content as unknown organic As. Till the end of the 1990s, iAs was assumed as the toxic actor among all the As species, and oAs was assumed less toxic or non-toxic. Thus, according to conventional opinion on the toxicity of As species, the negligible abundances of toxic As in *O. sinensis* show that the oral intake hazard might be lower than in iAs-accumulated mushrooms, such as *Laccaria amethystea* [30], *Collybia butyracea* [31] and other mushrooms [32]. However, this assumption was questioned with the development of analytic methods. Arsenic undergoes rapid and complicated metabolism in organisms, and several organic As species, discovered as intermediate metabolites (MMA^Ш^, DMA^Ш^, DMMTA^V^) in organisms or discovered in animal-sourced foods, were found to be strongly toxic (AsHCs) [15,16,17,18,19]. In this study, although the traditionally considered toxic As (iAs, DMA^V^, MMA^V^) in *O. sinensis* is minor, the large amount of organic As cannot be accurately determined for its speciation and thus its toxicity cannot be arbitrarily evaluated. In order to comprehensively assess long-term *O. sinensis* consumption, based on the intake of total As, i+uAs (considering uAs is toxic, as iAs), and iAs (consider uAs is non-toxic, as AsB) the average values of the estimated daily intake (*EDI)*, hazard quotient (*HQ*), and cancer risk (*CR)* were calculated according to Equations (1)–(3), respectively.

**Figure 6:**
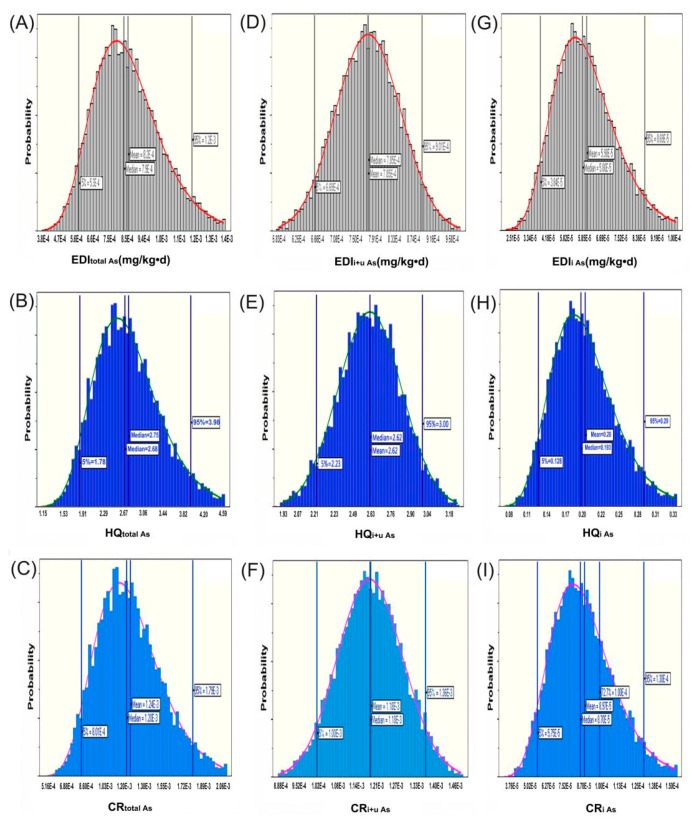
Estimated distribution patterns and descriptive statistics of (**A**,**D**,**G**) estimated daily intake (*EDI*), (**B**,**E**,**H**) hazard quotient (*HQ*), and (**C**,**F**,**I**) cancer risk (*CR*). Where in (**A**–**C**) values were derived according to the total As concentration in *O. sinensis*; in (**D**–**F**) values were derived according to the i+uAs concentration (the sum of iAs and uAs) in *O. sinensis*; and in (**G**–**I**) values were derived according to the iAs concentration in *O. sinensis*.

According to the intake of total As, the average of *HQ*_total As_ = 2.6 and *CR*_total As_ = 1.2 × 10^−3^. A Monte-Carlo simulation was used to model the uncertainties of the *EDI*, *HQ*, and *CR.* After 10,000 iterations of each simulation, *EDI*, *HQ*, and *CR* were derived, and the distribution patterns are illustrated in Figure 6. In terms of the health risk associated with total As exposure, the average *EDI*_total As_ (Figure 6A) from *O. sinensis* consumption is 8.00 × 10^−4^ mg/(kg·day), the *HQ*_total As_ > 1 (Figure 6B), and the mean, median, 5th percentile, and 95th percentile values of *CR*_total As_ are 1.23 × 10^−3^, 1.20 × 10^−3^, 8.14 × 10^−4^, and 1.78 × 10^−3^, respectively (Figure 6A–C)*.* According to the average value and uncertainty analysis of *EDI*, *HQ,* and *CR*, *O. sinensis* would not be recommended for long-time consumption, as assessed by the total As content (*HQ*_total As_ > 1 and *CR*_total As_ > 1 × 10^−4^). However, as we discussed before, applying total As content in the hazard risk assessment is unfair; the hazard risk assessment should be based on the concentration of toxic As species. However, the majority of the As species in *O. sinensis* were unknown using the current five arsenic standards, thus the toxicity is also unknown. Hence, we assess the edible hazard according to two postulated situations. First, if the uAs is as toxic as As^Ш^, based on the intake of i+uAs, *HQ*_i+uAs_ = 2.62 and *CR*_i+uAs_ = 1.2 × 10^−3^. In terms of the health risk associated with i+uAs exposure after the Monte-Carlo simulation, the mean, median, 5th percentile, and 95th percentile values of *CR*_i+u__As_ are 1.18 × 10^−3^, 1.18 × 10^−3^, 1.00 × 10^−^^3^, and 1.36 × 10^−3^, respectively (Figure 6D–F). These results indicate a similar edible hazard as that predicted by the total As analysis. Second, if the uAs is non-toxic as AsB and AsC, the relative abundance of the toxic iAs is only 10% of the total As content, and, and correspondingly, the average value (*EDI*_iAs_ = 5.95 × 10^−5^ mg/(kg·day), *HQ*_iAs_ = 0.19 < 1, and *CR*_iAs_ = 8.7 × 10^−5^ < 1 × 10^−4^) and uncertainty analysis of *EDI*, *HQ*, and *CR* were much lower and below the hazardous thresholds. The mean, median, 5th percentile, and 95th percentile values of *CR*_toxic As_ were 8.93 × 10^−5^, 8.66 × 10^−5^, 5.83 × 10^−5^, and 1.29 × 10^−4^, respectively (Figure 6G–I). The *CR* value exceeded the threshold value (1 × 10^−4^) at the 72.7th percentile. Thus, if the uAs is non-toxic, the long-term consumption of *O. sinensis* is relatively safe according to the hazard analysis on iAs and the reputation of *O. sinensis* should be rehabilitated because of its non-toxicity. As we discussed in Section 2.2, the uAs is might be an arsenosugar, which are generally considered to be non-toxic or have low toxicity [29]. Thus, according to the current recognition on the toxicity of organic As species, the uAs might be non-toxic, and accordingly the long consumption of *O. sinensis* might be safe in terms of As speciation analysis. However, determination of the structure and toxicity of the uAs should be carried out to provide strong evidence for our inference.

Although As has become notorious because some As species cause acute toxicity, chronic toxicity, and cancer via environmental exposure at low doses, its medicinal properties have been a focus in ancient China and Greece for over 3000 years [13]. Until the late 1940s, diseases such as psoriasis and syphilis were frequently treated with As [33]. More recently, inorganic arsenic has also shown significant activity in curing acute promyelocytic leukemia (APL) [13]. Inorganic arsenic has shown activity against other hematologic and solid organ malignancies but is also associated with serious toxicities, especially when applied at higher doses. For decades, scientists have attempted to seek ideal As compounds that are nontoxic and have medicinal potential. Few efforts have been successful, and the medical use of the most concerning forms of organic As (such as MMA^V^, DMA^V^, and AsB) has not been proven yet [33]. The recent development of orally bio-available organic arsenic derivatives (OAD) offering improved toxicity profiles and better efficacy may expand the application of arsenic compounds in hematologic malignancies and solid tumors [33,34]. *O. sinensis* has been massively reported to have predominant efficiency in anti-tumorigenesis, and most researchers consider this the result of chemical constituents such as cordycepin, adenosine, the exopolysaccharide fraction, cordyglucans, and monosaccharide saponins [5]. From our newly obtained data, inorganic As comprised only 7% of the total As content, much lower than that (90%) of unknown organic As. Inspired by the above-mentioned progress in the medicinal use of organic arsenic derivatives (OAD) [33,34], we infer that the unknown As might play certain important roles in anti-tumorigenesis or other functional uses of *O. sinensis*. Therefore, combining the risk hazard analysis and potential functional speculation, As might be considered functional in *O. sinensis* instead of poisonous. Further research to provide sufficient evidence on the above speculation including structural identification, toxicology, and pharmacology assessments, especially animal testing, should be implicated in future.

## 3. Materials and Methods

### 3.1. Reagents

Deionized water obtained from a Milli-Q water purification system (Millipore, Bedford, MA, USA) was used for the preparation of all solutions. Anhydrous sodium acetate, potassium nitrate, sodium dihydrogen phosphate, and disodium ethylenediaminetetraacetate (EDTA) were used for the preparation of mobile phases. H_2_O_2_ (31%) and concentrated HNO_3_ (69%) were used for sample digestion and extraction. Standards for the determination of both total As content and As speciation were prepared by diluting or compounding the standard stock solution sourced from the National Institute of Metrology (Beijing, China). The former was diluted to 10 mg As/L from the stock solution (As^V^) with certified concentration of 1000 mg As/L, and the latter was prepared with As^Ш^ (0.233 μmol/g), As^V^ (1.011 μmol/g), MMA^V^ (0.355 μmol/g), DMA^V^ (0.706 μmol/g), and AsB (0.518 μmol/g). All stock solutions were kept at 4 °C, and further dilutions for analysis were prepared daily. National Standard reference materials: GBW10049 (green Chinese onion, Institute of Geophysical and Geochemical Exploration, Langfang, China), GBW10051 (pork liver, Institute of Geophysical and Geochemical Exploration, Langfang, China), GBW08573 (yellow-fin tuna, Second Institute of Oceanography, Huangzhou, China) and GBW(E)100358 (rice, National Analysis Center for Ion and Steel, Beijing, China) were used as certified reference materials (CRMs) in this study.

### 3.2. Sample Collection and Preparation

Three typical production areas (Litang, Naqu, and Yushu) were chosen as the sampling sites (Figure 2), and twenty *O. sinensis* samples of similar size (approximately 0.3 g per sample) were collected at each site in May 2017. All the samples were mechanically cleaned of soil, rinsed with deionized water and freeze-dried to reach a constant weight. To gather enough material for subsequent As analysis, each of the five *O. sinensis* samples was combined into a batch sample. Therefore, the original twenty *O. sinensis* taken at each sampling site were organized into 4 sample batches (referred to as NQ1–4, LT1–4 and YS1–4). These sample batches were individually pulverized using a mortar and pestle to reduce the particle size. Then, the fine powder was passed through a sieve with a pore size of 0.25 mm and stored in sealable plastic bags at 4 °C until analysis.

For total As content analysis, digestion of approximately 500 mg of sample powder was conducted in a microwave digestion system using concentrated HNO_3_. The operating program of the microwave system was as follows: the samples were heated to 120 °C in 5 min and held at 120 °C for 5 min in the first step, heated to 150 °C in 5 min and held at 150 °C for 5 min in the second step, heated to 170 °C in 5 min and held at 170 °C for 5 min in the third step, and heated to 190 °C in 5 min and held at 190 °C for 20 min in the fourth step. Finally, the samples were cooled to room temperature. For As speciation analysis, approximately 1 g of sample powder was diluted with 20 mL 0.15 mol/L HNO_3_ in a 50 mL polyethylene centrifuge tube and then incubated in a 90 °C water bath for 12 h. All the digestion products were centrifuged, filtered, and stored at 4 °C before total As and As speciation analysis.

### 3.3. Total Arsenic Analysis

The total As content of each sample was measured using ICP-MS (Agilent 7800, Santa Clara, CA, USA). The supernatant was first diluted with water up to 25 mL and then subjected to ICP-MS. The operating parameters of the equipment were as follows: radio frequency (RF) power, 1550 W; carrier gas, 1.05 L/min; collision mode, Helium (HE) flow 4.2 mL/min; plasma gas flow rate = 15 L/min; auxiliary gas flow rate = 0.1 L/min; and selected isotope = *m*/*z* 75. Samples were quantified using an external calibration curve from As^V^ standards (calibration points: 5, 10, 50, 100, and 200 ppm). Triplicate analyses were performed for each sample. For quality control purposes, the standards used for the calibration curve were run before and after each sample series. The corresponding digestion blanks (one for each sample digestion series) were also measured. The total As in the CRMs (GBW10049, GBW10051, GBW08573 and GBW100358) were determined according the same methods.

### 3.4. Arsenic Speciation Analysis

The separation and determination of As species were performed according to our previous HPLC-ICP-MS method [35]. Five As species (As^Ш^, As^V^, MMA^V^, DMA^V^, AsB) were separated by an Agilent 1260 HPLC system (Agilent, USA). The chromatograph was equipped with a standard autosampler, IonPac AG19 guard column (4 × 50 mm), and an IonPacAS19 separation column (4 × 250 mm). The main chromatography conditions used for HPLC were as follows: a mobile phase of 10 mmol/L anhydrous sodium acetate; 3 mmol/L potassium nitrate; 10 mmol/L sodium dihydrogen phosphate; 0.2 mmol/L disodium ethylenediaminetetraacetate buffer; a flow rate at 1.0 mL/min; a column temperature of room temperature; and an injection volume with a 50 μL sample. The separated As species were examined by ICP-MS (as described previously) and identified by comparison of retention times with standards (Figure 4A). External calibration curves (calibration points: 0, 2.5, 5, 10, 50, and 100 ppb) were used to quantify MMA^V^, DMA^V^, As^Ш^, As^V^, and AB according to the corresponding standards. Triplicate analyses were performed for each sample. Extraction blanks were also analyzed by HPLC-ICP-MS in each work session.

To check the extraction and digestion efficiency for the As species analysis, the As species extraction was completely measured using ICP-MS according to 3.3. Then the measured total As concentration in the As species extraction which was digested by 0.15 mol/L HNO_3_, was compared with the total As digested by concentrated HNO_3_ to calculate the extraction efficiency. The iAs species in one CRM (GBW100358) were determined according the same methods.

Due to the complexity of the metrics of the *O. sinensis* sample, the As^Ш^ peak could not be separated from the other unknown peaks (Figure 4B); therefore, 1 mL of H_2_O_2_ was added to the supernatant before analysis. This operation completely oxidizes As^Ш^ into As^V^ (Figure 4C), without conversion of other arsenic species into new As compounds, according to the comparison of the number and the area of the corresponding peaks in Figure 4B and C. The inorganic As content was identified and quantified as As^V^ using external standards. The total organic As amounts were quantified according to the delta value between the total As and the inorganic As content. Thus, the content of the unknown As species was quantified according to the delta value between the organic As and the sum of the three determined As species (MMA^V^, DMA^V^, and AsB).

### 3.5. Health Risk Assessment

The health risks for consumers with exposure to total toxic As (the sum of inorganic As, DMA, and MMA^V^), total As content, i+uAs, and iAs in *O. sinensis* were evaluated. According to the model of evaluation for health risks (Environment Protection Agency, EPA) [25,26], the estimated daily intake of As from *O. sinensis* consumption was calculated according to Equation (1):

(1) EDI=CAs×IRBW,

where *EDI* (mg/kg·day) is the estimated daily intake of As, *C_As_* (mg/kg) is the concentration of total As or toxic As in *O. sinensis* (dry weight), *IR* (0.01 kg/day) is the daily ingestion rate of *O. sinensis* [36], and *BW* is the average body weight (58.7 kg) [37] of the consumer.

Risk characterization was quantified according to the potential non-carcinogenic risks of As exposure from *O. sinensis* ingestion and was reflected by the hazard quotient (*HQ*). The *HQ* value was calculated using equation (2). If *HQ* < 1, there might be no concern for non-carcinogenic effects. Otherwise, if the *HQ* exceeds 1, there might be serious concern for non-carcinogenic effects [25].

(2) HQ=(EDIRfD)×(EF×EDAT)

The probability of the excess lifetime cancer risk (*CR*) due to As exposure for *O. sinensis* consumption was estimated according to Equation (3). According to the US EPA, the incremental probability of cancer risk for consumers over a lifetime is characterized by *CR* with an acceptable range of 1 × 10^−6^–1 × 10^−4^. A *CR* less than 1.0 × 10^−6^ indicates no obvious concern for cancer risk. However, a *CR* greater than 1.0 × 10^−4^ indicates an obvious potential cancer risk [25].

(3) CR=EDI×SF×(EF×EDAT)

In Equations (2) and (3), *RfD* is the oral reference dose for As, *EF* (days/year) is the exposure frequency, *ED* (years) is the exposure duration, *AT* (days) is the average exposure time to As (usually *AT* is equal to the average life expectancy), and *SF* is the slope factor of As through oral intake. In this study, *RfD*, *SF*, *ED*, *EF*, and *AT* were determined to be 3 × 10^−4^ mg/(kg·day) [26], 1.5 mg/(kg·day) [26], 70 years, 365 days/year, and 25,550 days [37], respectively.

To assess the long-term health risks of *O. sinensis* to arsenic exposure, the uncertainty of *EDI*, *HQ*, and *CR* were modeled through a Monte-Carlo simulation conducted with CrystalBall Software (V. 11.1.2.0.00, Oracle, Redwood Shores, CA, USA). The parameter distributions used in the model are presented in Table A1.

## 4. Patents

A new method for risk assessment of *Ophiocordyceps sinensis*. Patent No. 201711087936.2. Document No. 2017110900896410.

## Figures and Tables

**Table 1:** Analytical performance of inductively coupled plasma mass spectrometry (ICP-MS) for total arsenic content and HPLC-ICP-MS for arsenic species.

Analytes	Linear Range (μg/L)	Linear Equation	*R*	LOD (μg/kg)	LOQ (μg/kg)
Total As	0.5–500	y = 2731.03x + 5.5567	0.9998	2.3	6.9
AsB	0.2–300	y = 18,867.9x + 2319.6	0.9999	1.1	3.3
DMA^V^	0.2–300	y = 19,212.4x + 4065.2	0.9999	1.3	4.0
As^Ш^	0.2–300	y = 17,090.6x + 3289.1	0.9997	1.0	3.0
MMA^V^	0.5–300	y = 18,373.6x + 196.8	1.0000	2.2	6.6
As^V^	0.2–300	y = 18,504.6x + 8247.2	1.0000	1.1	3.3

**Table 2:** Recovery and precision of the methods.

Analytes	Background Value (mg/kg)	Added (μg/L)	Measured Value (μg/L)	Recovery (%)	RSD (%, *n* = 6)
Total As	0.51	5.00	14.8~15.3	92.3~99.4	4.6
		10.0	20.4~21.5	94.7~102.3	3.8
		50.0	60.7~64.3	95.8~106.6	2.4
AsB	0.010	2.00	2.09~2.26	91.7~100.1	4.2
		10.0	9.45~9.87	91.9~96.2	1.9
		50.0	42.0~47.0	83.6~93.4	5.0
DMA^V^	ND	2.00	1.73~1.98	86.5~99.0	5.7
		10.0	9.20~9.55	92.0~95.5	1.5
		50.0	50.4~52.6	100.9~105.2	1.7
As^Ш^	1.70	2.00	45.3~45.6	86.3~99.2	5.5
		10.0	52.8~53.7	88.8~98.0	4.3
		50.0	96.8~99.2	106.9~111.7	2.0
MMA^V^	0.0031	2.00	2.07~2.19	99.4~105.7	2.7
		10.0	10.6~10.8	105.0~107.2	0.85
		50.0	47.2~48.6	94.2~97.1	1.2
As^V^	0.12	2.00	4.81~5.08	86.6~100.0	6.6
		10.0	12.8~13.0	97.4~99.4	0.80
		50.0	49.0~50.2	91.9~94.4	1.1

**Table 3:** National standard reference materials values (mg/kg, mean ± standard deviation) and determined values for total As and inorganic arsenic (iAs) content (*n* = 5).

Sample Type	Reference Materials	Certified Value (mg/kg)	Determined Value (mg/kg)	Recovery (%)
Green Chinese onion	GBW10049	0.52 ± 0.11	0.507 ± 0.08	97.5
Pork liver	GBW10051	1.4 ± 0.3	1.42 ± 0.15	101.4
Yellow-fin tuna	GBW08573	5.08 ± 0.39	4.98 ± 0.11	98.0
Rice	GBW100358	0.16 ± 0.02 (total As)	0.165 ± 0.012	103.1
		0.13 ± 0.02 (iAs)	0.144 ± 0.006	110.8

**Table 4:** Concentration ^a^ of total arsenic and arsenic species in *Ophiocordyceps sinensis.*

Sample Name	AsB ^b^ mg/kg (%)	DMA^V^	MMA^V^	uAs mg/kg (%)	As^Ш^ mg/kg (%)	As^V^ mg/kg (%)	iAs mg/kg (%)	oAs mg/kg (%)	Total As mg/kg
NQ1	0.10 (2.1%)	nd ^c^	nd	4.34 (91.2%)	0.22 (4.6%)	0.10 (2.1%)	0.32 (6.7%)	4.44 (93.3%)	4.76
NQ2	0.09 (1.8%)	nd	nd	4.59 (91.8%)	0.21 (4.2%)	0.11 (2.2%)	0.32 (6.4%)	4.68 (93.6%)	5.00
NQ3	0.12 (2.3%)	nd	nd	4.81(91.6%)	0.25 (4.8%)	0.07 (1.3%)	0.32 (6.1%)	4.93 (93.9%)	5.25
NQ4	0.08 (2.0%)	nd	nd	3.69 (90.0%)	0.23 (5.6%)	0.10 (2.4%)	0.33 (8.0%)	3.77 (92.0%)	4.10
LT1	0.07 (1.7%)	nd	nd	3.69 (90.2%)	0.20 (4.9%)	0.13 (3.2%)	0.33 (8.1%)	3.76 (91.9%)	4.09
LT2	0.09 (2.2%)	nd	nd	3.72 (89.6%)	0.22 (5.3%)	0.12 (2.9%)	0.34 (8.2%)	3.81 (91.8%)	4.15
LT3	0.11 (2.8%)	nd	nd	3.56 (89.0%)	0.24 (6.0%)	0.09 (2.3%)	0.33 (8.3%)	3.67 (91.7%)	4.00
LT4	0.09 (1.7%)	nd	nd	4.75 (92.2%)	0.21 (4.1%)	0.10 (1.9%)	0.31 (6.0%)	4.84 (94.0%)	5.15
YS1	0.12 (2.6%)	nd	nd	4.19 (89.3%)	0.27 (5.8%)	0.11 (2.3%)	0.38 (8.1%)	4.31 (91.9%)	4.69
YS2	0.08 (1.9%)	nd	nd	3.80 (90.5%)	0.24 (5.7%)	0.08 (1.9%)	0.32 (7.6%)	3.88 (92.4%)	4.20
YS3	0.07 (1.4%)	nd	nd	4.69 (92.3%)	0.25 (4.9%)	0.07 (1.4%)	0.32 (6.3%)	4.76 (93.7%)	5.08
YS4	0.15 (2.9%)	nd	nd	4.65 (90.5%)	0.22 (4.3%)	0.12 (2.3%)	0.34 (6.6%)	4.8 (93.4%)	5.14
AVR ^d^	0.09 (1.9%)	nd	nd	4.21 (90.9%)	0.23 (5.0%)	0.10 (2.2%)	0.33 (7.1%)	4.3 (92.9%)	4.63

^a^ Concentrations are presented as the average value of three measurements with a relative standard deviation (RSD) of less than 8% (the ranges of RSD values were as follows, RSD_AsB_: 2.1~5.6%, RSD_DMA__V_: 1.8~6.1%, RSD_As__Ш_: 2.0~6.7%, RSD_MMA__V_: 1.4~5.2%, and RSD_As__V_: 1.1~7.3%; ^b^ AsB, MMA, DMA, uAs, AsⅢ, AsⅤ, iAs, oAs, and Total As were the abbreviation of arsenobetaine, monomethylarsonic acid, dimethylarsenic acid, unknown organic arsenic, arsenite, and arsenate, inorganic arsenic (total), organic arsenic (total), and total arsenic, respectively. ^c^ not detected; ^d^ average value among all the samples.

## Abbreviations

*O. sinensis*	*Ophiocordyceps sinensis*
As	Arsenic
As^Ш^	Arsenite
As^V^	Arsenate
DMA	Dimethylarsinic acid
MMA	Methylarsonic acid
AsB	Arsenobetaine
DMMTA	Thio-organoarsenic
uAs	Unknown organic As
oAs	Organic arsenic
iAs	Inorganic arsenic
i+uAs	Sum of iAs and uAs
LOQ	Limits of quantification
LOD	Limits of detection
*EDI*	Estimated Daily Intake
*HQ*	Hazard Quotient
*CR*	Cancer Risk
HPLC-ICP-MS	High-Performance Liquid Chromatography–Inductively Coupled Plasma Mass Spectrometry

## Appendix A

**Table A1:** Input parameters for Monte-Carlo simulation.

Parameters	Units	Distribution
Adult Body weight, BW	BW kg	Lognormal (Mean = 58.7, SD = 12.0)
Daily ingestion rate, *IR*	kg/day	Lognormal (Mean = 0.01, SD = 0.0143)
Total As concentration, *C*_total As_	μg/kg	Lognormal (Mean = 4.63, SD = 0.42)
i+uAs concentration, *C*_i+uAs_	μg/kg	Lognormal (Mean = 4.53, SD = 0.41)
iAs concentration, *C*_iAs_	μg/kg	Lognormal (Mean = 0.34, SD = 0.03)
